# The NAC-type transcription factor *OsNAC2* regulates ABA-dependent genes and abiotic stress tolerance in rice

**DOI:** 10.1038/srep40641

**Published:** 2017-01-11

**Authors:** Jiabin Shen, Bo Lv, Liqiong Luo, Jianmei He, Chanjuan Mao, Dandan Xi, Feng Ming

**Affiliations:** 1 State Key Laboratory of Genetic Engineering, Institute of Genetics, Institute of Plant Biology, School of Life Science, Fudan University, Shanghai 200433, China; 2 Rice Research Institute, Sichuan Agricultural University, Sichuan 611130, China

## Abstract

Plants can perceive environmental changes and respond to external stressors. Here, we show that *OsNAC2*, a member of the NAC transcription factor family, was strongly induced by ABA and osmotic stressors such as drought and high salt. With reduced yields under drought conditions at the flowering stage, *OsNAC2* overexpression lines had lower resistance to high salt and drought conditions. RNAi plants showed enhanced tolerance to high salinity and drought stress at both the vegetative and flowering stages. Furthermore, RNAi plants had improved yields after drought stress. A microarray assay indicated that many ABA-dependent stress-related genes were down-regulated in *OsNAC2* overexpression lines. We further confirmed that *OsNAC2* directly binds the promoters of *LATE EMBRYOGENESIS ABUNDANT 3 (OsLEA3*) and *Stress-Activated Protein Kinases 1 (OsSAPK1*), two marker genes in the abiotic stress and ABA response pathways, respectively. Our results suggest that in rice *OsNAC2* regulates both abiotic stress responses and ABA-mediated responses, and acts at the junction between the ABA and abiotic stress pathways.

Drought, high salinity, and low temperature are major stress factors affecting plant growth^1^. When plants are exposed to variable environments, cells perceive stress signals from the outside world. This process occurs through a series of complex signalling pathways including the ABA-dependent and ABA-independent pathways^2^,^3^. The stress signals are sent to transcription factors (TFs) involved in stress responses that then trigger the expression of downstream stress response genes. Through this mechanism, plants reduce the negative influence of abiotic stressors by activating stress tolerance reactions^4^.

Members of the NAC TF family, which is specific to higher plants, can bind to promoter DNA as a dimer and induce gene expression. The name is derived from the first letters of three genes, *NAM* from a petunia hybrid and *ATAF1/2* and *CUC2* from *Arabidopsis thaliana,* that were initially found by Souer and Aida^5^,^6^. To date, there are 117 and 151 NAC TFs in *Arabidopsis* and *Oryza sativa* (rice), respectively^7^. Structural and functional analyses indicated that the NAC family is paralogous to the plant WRKY TF family^8^. Further evolutionary analysis showed that ancient eukaryotic WRKY proteins may be the common ancestors of plant NAC and WRKY TFs and animal GCM TFs^9^. NAC proteins have a highly conserved N-terminal domain called the NAC domain that is responsible for DNA binding and a variable C-terminal domain that is a transcriptional activation domain^10^.

NAC family TFs are mainly involved in plant growth, development, and biotic or abiotic stress responses. The petunia *NAM* gene is required for shoot tip meristem formation^5^ while *Arabidopsis CUC1*^11^, *CUC2*^6^, *CUC*^12^, and rice *OsNAC2*^13^ can inhibit the growth of certain cells, promote the production of axillary meristems, and promote the development of organ boundaries. *NAC1*^14^ and *AtNAC2*^15^ are induced by IAA and promote lateral root growth and *NST1* and *NST2* co-regulate *Arabidopsis* secondary cell wall synthesis^16^,^17^,^18^. *SND1* stimulates the expression of secondary cell wall synthesis genes^19^ while *VND6/7* are key genes regulating *Arabidopsis* xylem development^20^. NAC family members also play important roles in cell division and extension^21^, floral development and flowering^22^, senescence^23^,^24^,^25^, and seed germination^26^,^27^.

Studies have shown that abiotic stressors can induce the expression of many rice NAC TF genes. *SNAC1* can greatly increase rice drought tolerance in both the vegetative and flowering stages^28^. OsNAC5^29^ and OsNAC6^30^,^31^ proteins can bind to the promoter of *OsLEA3* and significantly increase high salt and drought tolerance. Overexpression of *OsNAC10* increased rice yield under drought conditions^32^. *ANAC019, ANAC055* and *ANAC072* are induced by drought, high temperatures, and ABA and are required for drought tolerance^33^. Transgenic *Arabidopsis* plants expressing the rice gene *OsNAC063* had enhanced high salt and drought tolerance^34^ while *ATAF1* may negatively control functional genes in drought stress^35^. Transgenic *Arabidopsis* plants expressing *LOV1* had enhanced resistance to low temperatures^36^ while the overexpression of *ANAC102* in *Arabidopsis* confers resistance to hypoxic stress^37^. No reports have, however, been published outlining the role of *OsNAC2* in abiotic stress responses.

In an earlier study, overexpression of *OsNAC2* in rice was reported to increase the tiller number^13^. Our lab previously found that *OsNAC2* was also involved in the regulation of plant height through the GA pathway^38^. Here, we have shown that *OsNAC2* is induced by ABA and osmotic stressors like drought and high salt. Rice *OsNAC2* overexpression lines had lower drought and high salinity tolerance in both the vegetative and flowering stages compared with wild-type (WT) plants, while in RNAi lines have higher drought and high salinity resistance performance. Additionally, RNAi plants maintained high yields under drought conditions. Gene expression analysis showed that *OsNAC2* overexpression down-regulated ABA-dependent stress-related marker genes, suggesting that *OsNAC2* is a negative regulator of the high salinity and drought response pathways. We further explored OsNAC2 target genes using ChIP and yeast-one-hybrid analyses. Our data suggests that OsNAC2 has a new function and regulation mechanism in abiotic stress responses by directly regulating *OsLEA3 (LATE EMBRYOGENESIS ABUNDANT 3*) and *OsSAPK1 (Stress-Activated Protein Kinases 1*). It may, therefore, play an important role in linking the ABA and abiotic stress response pathways together.

## Results

### *OsNAC2* expression is induced by osmotic stress and ABA

**Figure 1:**
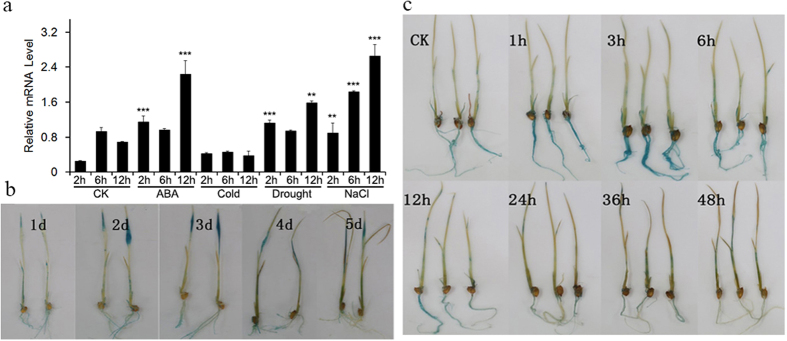
Expression induction of *OsNAC2* under different stress and hormone treatments. (**a**) Quantitative polymerase chain reaction (PCR) analysis of *OsNAC2* expression in response to ABA (100 mM), cold, drought (20% PEG8000) and NaCl (200 mM) for 2 h, 6 h and 12 h. RNA was extracted from whole seedlings. Data were means ± SE with at least three replicates. Asterisks represent statistically significant differences between CK and treated lines. **P < 0.01, ***P < 0.001. (**b**) GUS staining of *Pro*_*OsNAC2*_:GUS seedlings under NaCl (150 mM) treatment for 1d, 2d, 3d, 4d and 5d. (**c**) GUS staining of *Pro*_*OsNAC2*_:GUS seedlings under air dry treatment from 0 h to 2d.

Expression of *OsNAC2* in response to ABA, low temperature, dehydration, and NaCl was analysed using qRT-PCR to show time-dependent induction patterns. The *OsNAC2* transcript accumulated within 2 hours under ABA, dehydration, and NaCl treatments, with peak expression reached after 12 hours. Conversely, expression of *OsNAC2* decreased slightly during 12 hours of low-temperature treatment (Fig. 1a). There was no apparent pattern in *OsNAC2* mRNA accumulation in plants treated with water only.

To study the effect of the promoter region on the expression of *OsNAC2* in seedlings under abiotic stress, we generated transgenic rice plants containing a 1500-bp *OsNAC2* promoter fragment (Fig. S1) fused with a *GUS* reporter gene to visualise is localisation (Fig. S2). Histochemical GUS staining indicated that the blue colouration was deepest in the leaves of seedlings treated with NaCl for 2d (Fig. 1b) and in the roots of seedlings air-dried for 3 h (Fig. 1c). With both treatments, the expression subsequently decreased as the treatment time increased. The results indicated that *OsNAC2* will be induced by NaCl and mainly expressed in leaf. However, *OsNAC2* was induced by drought treatment and expressed mostly in root. This suggests that *OsNAC2* might play a role in different responses to various abiotic stressors.

### Changes in *OsNAC2* expression affects salt sensitivity

**Figure 2:**
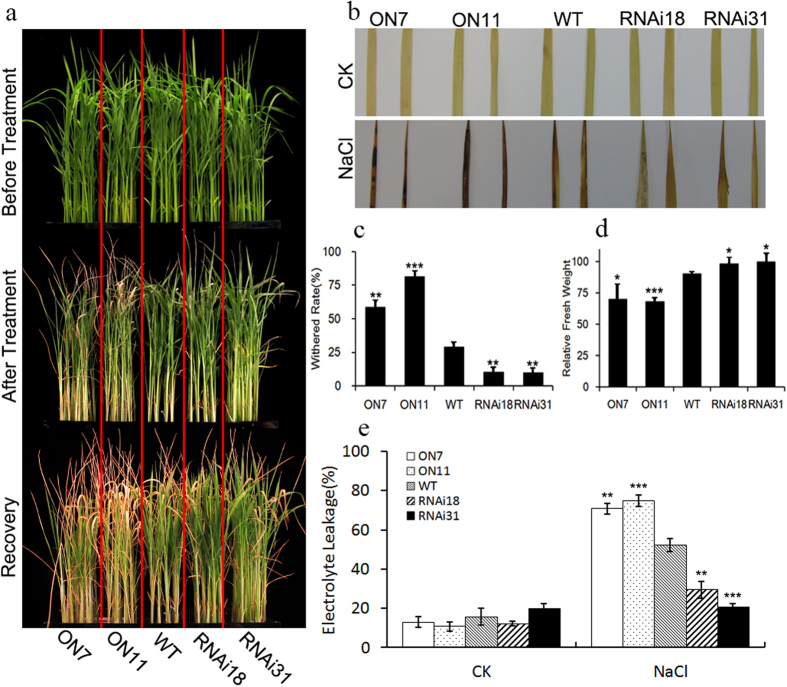
Phenotype analysis of *OsNAC2* transgenic plants and WT in response to 150 mM NaCl treatment. The seedlings were cultivated in basal nutrient solution and normal conditions, 28 °C, 16 h light and 8 h dark. Then two-week-old seedlings were transferred into nutrient solution containing 150 mM NaCl for 2.5d and recovery for 2d. (**a**) Phenotype of WT and *OsNAC2* transgenic plants before and after high salt (150 mM NaCl) treatment for 2.5d and recovery for 2d. (**b**) DAB staining of WT and transgenic seedlings leaves after recovery. (**c**) Withered rate of WT and transgenic seedlings after recovery. (**d**) Relative fresh weight of WT and transgenic seedlings after recovery. (**e**) Electrolyte leakage of WT and transgenic seedlings after recovery. Data were means ± SE with at least five replicates. Asterisks represent statistically significant differences between WT and transgenic plants. *P < 0.05, **P < 0.01, ***P < 0.001.

Given that *OsNAC2* expression was induced by salt and drought stress, we designed a set of experiments to test its function in abiotic stress tolerance. Therefore, ON7 (*OsNAC2* overexpression line no. 7), ON11 (*OsNAC2* overexpression line no. 11), RNAi18 (*OsNAC2* RNAi line no. 18), RNAi31 (*OsNAC2* RNAi line no. 31), and WT plants were evaluated for high salinity tolerance and drought stress. In *OsNAC2* overexpression lines, OsNAC2 is promoted by 35 S, and fused with GFP-tag. Two-week-old rice seedlings were treated with 150 mM NaCl for 2.5 d. After high salt treatment, plants were transferred to recover in normal water for 2 d. All the plants grew well under normal conditions. After high salt treatment and recovery, ON7 and ON11 plants were severely withered and had damaged leaves (Fig. 2a). Compared with the WT, significantly more seedlings of the ON7 and ON11 overexpression lines withered (58.5% and 81.3%. respectively). Of the RNAi18 and RNAi31 seedlings, 10.4% and 9.8% withered, respectively, whereas 29.0% of the WT seedlings withered (Fig. 2c). We used diaminobenzidine (DAB) staining to test reactive oxygen species in all the plant lines. Under normal conditions (CK), there was no DAB staining in WT and transgenic plants. After high salt treatment, DAB staining accumulated in ON7 and ON11 leaves and, to a lesser degree, in the leaves of RNAi and WT seedlings (Fig. 2b). These results indicated greater ROS accumulation in overexpression lines after high salinity stress. We also evaluated the effect of salt stress by examining electrolyte leakage and relative fresh weight. All the plants had low electrolyte leakage under normal growth conditions. Overexpression plants exhibited a significantly lower relative fresh weight than WT plants while RNAi18 and RNAi31 had significantly higher fresh weights compared with WT (Fig. 2d). At the same time, a greater increase in electrolyte leakage was found in ON7 and ON11 plants than in WT plants after 150 mM NaCl treatment, while RNAi18 and RNAi31 plants had markedly smaller increases in electrolyte leakage than WT (Fig. 2e). These results indicated that overexpression of OsNAC2 suppresses salt resistance in transgenic plant.

**Figure 3:**
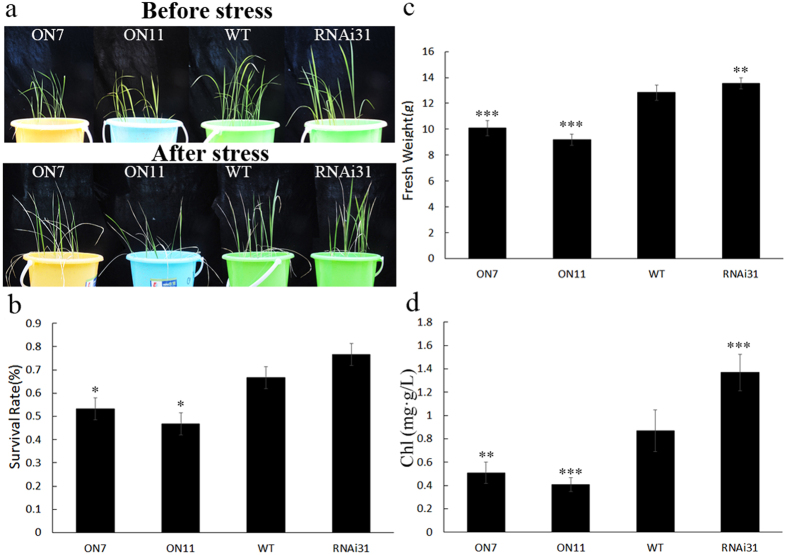
Soil experiment analysis of high salt stress in control and transgenic rice plants. Rice seedlings were grown under normal conditions for four weeks, and then irrigated with 150 mM NaCl solution for 14d with 7d recovery. (**a**) Phenotype of WT and *OsNAC2* transgenic plants before and after high salt (150 mM NaCl) stress for 14d in pot. (**b**) Survival rate of WT and transgenic seedlings after stress. (**c**) Fresh weight of WT and transgenic seedlings after high stress. (**d**) Chlorophyll content of WT and transgenic seedlings after high stress. The results are averages of three independent experiments with 10 plants per experiment. Data were means ± SE. Asterisks represent statistically significant differences between WT and transgenic plants. *P < 0.05, **P < 0.01, ***P < 0.001.

Compared with hydroponics, growing plants in pots of soil more closely resembles the real growing environment of rice. All the rice seedlings were, therefore, grown hydroponically until they were 4 w old before being transferred into pots for soil experiments. After 14 d of 150 mM NaCl treatment, the leaves of ON7 and ON11 plants were yellow and wilting while the leaves of RNAi31 plants remained green and upright (Fig. 3a). Approximately 50% of ON7 and ON11 plants had died by this point while approximately 60% of the corresponding WT plants and over 70% of the RNAi31 plants remained alive (Fig. 3a). We also evaluated the viability of all the plants by measuring fresh weights. Before NaCl treatment, there were no significant differences in the fresh weight of WT and transgenic rice seedlings under normal soil conditions. After high salt stress, ON11 lines lost more fresh weight than the corresponding WT plants while RNAi31 lost less fresh weight (Fig. 3c). Chlorophyll plays an important role in plant growth and development. After salt stress, more chlorophyll was degraded in ON11 than in the WT plants while more chlorophyll was conserved in the RNAi31 plants compared with WT (Fig. 3d). These data demonstrated that overexpression of *OsNAC2* could make rice more sensitive to high salt stress whether in hydroponic or soil culture conditions.

### Changes in *OsNAC2* expression affect drought sensitivity

**Figure 4:**
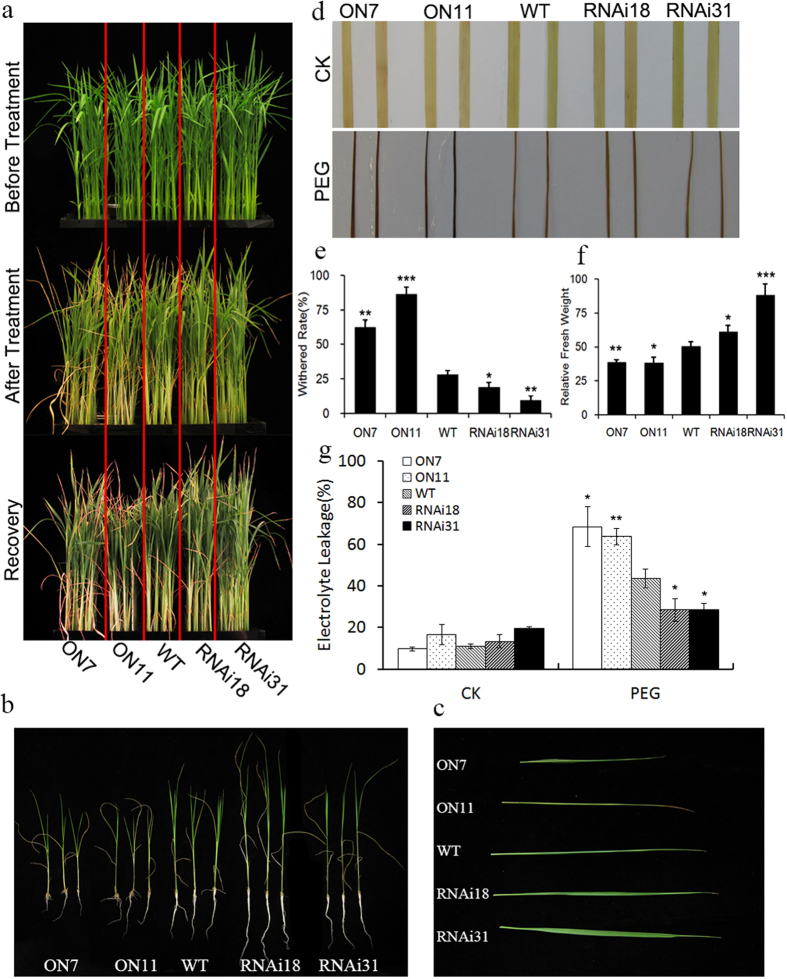
Phenotype analysis of *OsNAC2* transgenic plants and WT response to 20% PEG8000 treatment. The seedlings were cultivated in basal nutrient solution and normal conditions, 28 °C, 16 h light and 8 h dark. Then two-week-old seedlings were transferred into nutrient solution containing 20% PEG8000 for 5d and recovery for 3d. (**a**) Phenotype of WT and *OsNAC2* transgenic plants before and after PEG8000 (20%) stress for 5d and recovery for 3d. (**b**,**c**) Phenotype of ON7, ON11, WT, RNAi18 and RNAi31 after salt treatment. Leaves turned yellow and rolled into a needle-like shape. (**d**) DAB staining of WT and transgenic seedlings leaves after recovery. (**e**) Withered rate of WT and transgenic seedlings after recovery. We sampled 96 individuals of each line. Then we measured the length of all leaves and the length of withered parts in each line, and calculated the result by withered parts’ length/Total leaves length. The assay was repeated three times. (**f**) Relative fresh weight of WT and transgenic seedlings after recovery. (**g**) Electrolyte leakage of WT and transgenic seedlings after recovery. Data were means ± SE with at least three biological replicates. Asterisks represent statistically significant differences between WT and transgenic plants. *P < 0.05, **P < 0.001, ***P < 0.001.

As with salinity stress, *OsNAC2* transcription was also induced by drought treatment (Fig. 1a), suggesting that *OsNAC2* might also play an important role in the response to drought stress. We used PEG8000 to mimic natural drought conditions. Two-week-old transgenic and WT rice seedlings were transferred into nutrient solution with 20% PEG8000. After 5d treatment and 3d recovery, more leaves of the overexpression lines ON7 and ON11 (62.5% and 86.5%, respectively) had rolled into a needle-like shape than the WT (28.1%), while less RNAi18 and RNAi31 plants had turned yellow (18.8% and 9.4%, respectively) but no needle-like shape leaves (Fig. 4a,b,c,e). DAB staining and analysis of the relative fresh weight and electrolyte leakage were performed to evaluate the drought resistant phenotype. Greater ROS accumulation was observed in ON7 and ON11 lines (Fig. 4d) and both lost more fresh weight than the WT plants during PEG treatment and had significantly more electrolyte leakage (Fig. 4f,g).

**Figure 5:**
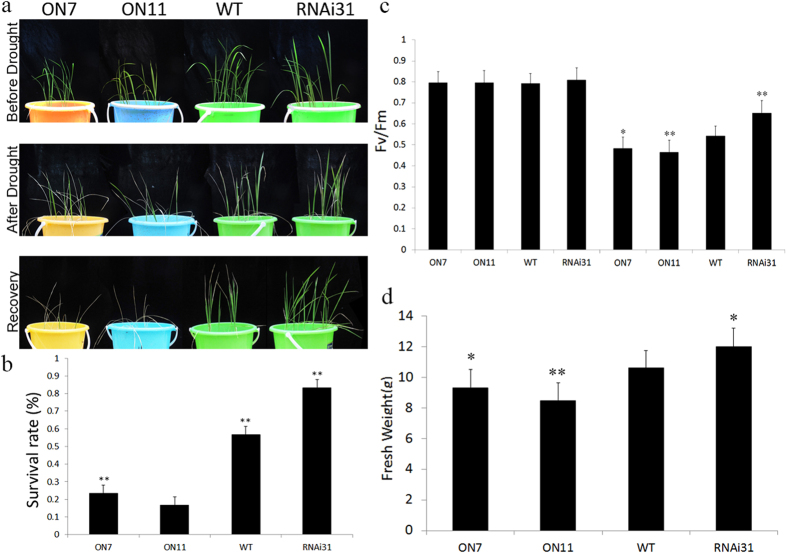
Phenotype analysis of *OsNAC2* transgenic plants and WT in response to drought treatment for 14 days in the soil. Rice seedlings were grown under normal conditions for four weeks, gradually reduced water supply and cut out water for 14d with 7d recovery. (**a**) Phenotype of WT and *OsNAC2* transgenic plants before and after drought stress, and after 7d recovery. (**b**) Survival rate of WT and transgenic seedlings after recovery. (**c**) Changes in Chl fluorescence (F_v_/F_m_) under normal conditions and drought treatment. (**d**) Overground fresh weight of WT and transgenic seedlings after recovery. The results are averages of three independent experiments with 10 plants per experiment. Data were means ± SE with at least three biological replicates. Asterisks represent statistically significant differences between WT and transgenic plants. *P < 0.05, **P < 0.01, ***P < 0.001.

Our results confirmed that overexpression of *OsNAC2* decreased the tolerance of rice to the PEG solution leading us to assume that changes in *OsNAC2* expression would affect the drought sensitivity of rice. To evaluate the drought tolerance of transgenic and WT rice seedlings we used four-week-old transgenic seedlings that were grown in pots alongside corresponding WT plants. In normal conditions, no phenotypic differences were observed and each seedling was healthy. After 14 d without watering, ON7 and ON11 seedlings turned yellow and wilted while RNAi31 plants remained green. These differences became more obvious after 7 d of re-watering (Fig. 5a). The survival rate of ON7 and ON11 significantly decreased compared with the WT, while the survival rate of RNAi31 increased significantly (Fig. 5b). The F_v_/F_m_ value, which represents the activity of PSII, was used to evaluate the extent of rice plant damage, with more damaged plants having lower values. The F_v_/F_m_ values were lower in ON11 than in the WT, while the values were higher in RNAi31 (Fig. 5c). The post-treatment fresh weights correlated with the *OsNAC2* transcript level; ON7 and ON11 had lower fresh weights than the WT while RNAi31 plants had higher values (Fig. 5d). These data indicated that overexpression of *OsNAC2* could make rice plants more sensitive to drought stress at the vegetative stage.

**Figure 6:**
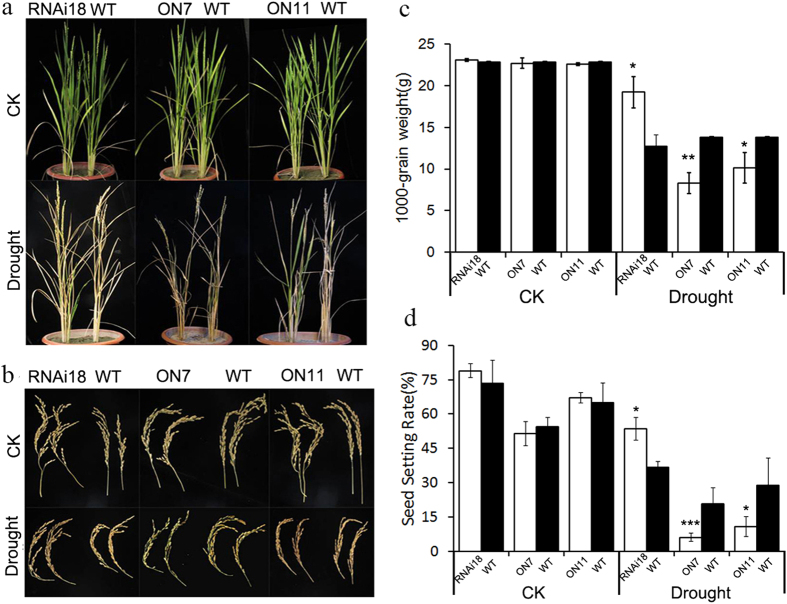
Drought tolerance of *OsNAC2* transgenic rice at the flowering stage. (**a**) Phenotype of reproductive rice under normal and drought conditions. (**b**) The spikelet of rice plants under normal and drought conditions. (**c**) 1000-grain weight of rice plants under normal and drought conditions. (**d**) Seed setting rate of rice plants under normal and drought conditions. We counted full seeds and empty seeds proportion in 300 grains of each line, and calculated by full seeds/300. The assay was repeated three times.

As rice yields and phenotypes are hypersensitive to drought stress during flower development^39^, we tested the drought tolerance of transgenic rice at this stage. All the seedlings were grown in pots under normal growth conditions before they reached the heading stage. Control pots were watered regularly while those of drought treatment groups were not irrigated for 12 d until harvest. Phenotypic analysis of the different lines after water deficient and recovery shown that ON7 and ON11 suffered severe growth retardation and high senescence (Fig. 6a). We measured several agronomic traits to evaluate the drought damage to rice. After drought treatment, ON7 and ON11 plants had the lowest 1000-grain weight, while RNAi18 plants had the highest (Fig. 6b,c). The seed setting rate of ON7 and ON11 was also visibly lower than in WT while RNAi18 plants had a significantly higher seed setting rate (Fig. 6d). These results further confirmed that overexpression of *OsNAC2* made rice plants hypersensitive to drought tolerance and decreased their yield under drought stress.

### Stress-related marker genes are all down-regulated in *OsNAC2* overexpression plants

**Figure 7:**
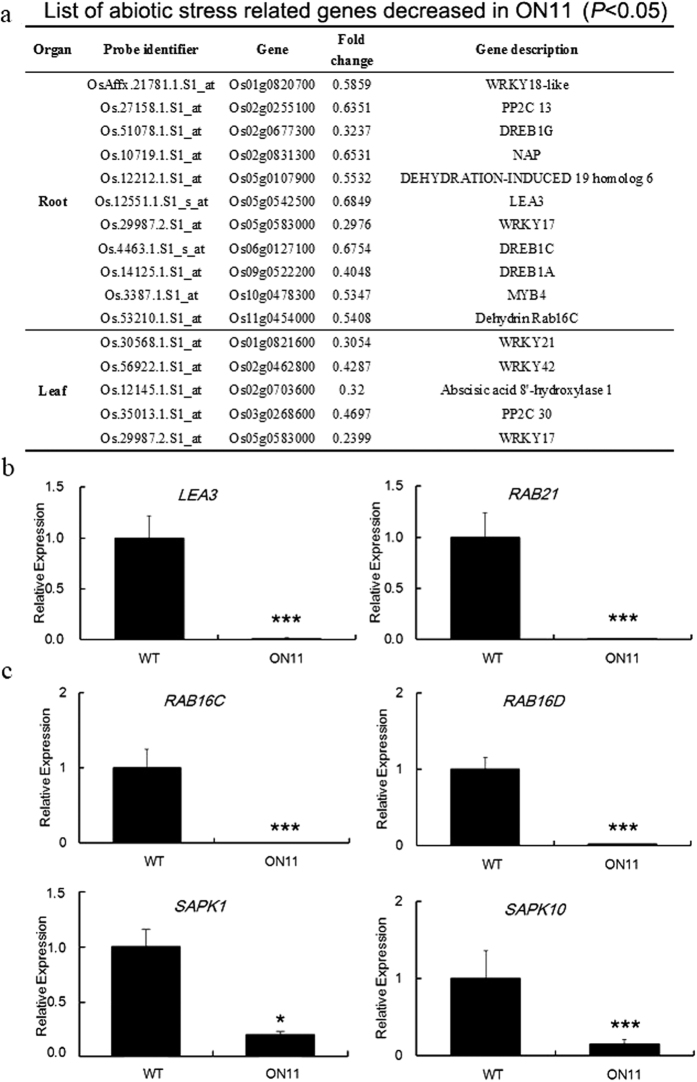
Microarray analysis and real time PCR confirmation of abiotic stress related genes. (**a**) List of abiotic stress related genes which decreased in ON11. (**b**,**c**) Expression of stress related genes in leaf and root respectively. Data were means ± SE with at least three biological replicates. Asterisks represent statistically significant differences between WT and transgenic plants. *P < 0.05, **P < 0.01, ***P < 0.001.

The above results showed that overexpression of OsNAC2 reduced plant tolerances to drought and high salinity stress. To further explore the regulation mechanism of OsNAC2 in the abiotic stress response pathway, we performed gene expression profiling analysis compared WT and *OsNAC2*-overexpressing plants using the existing rice microarray data in our lab. Compared with WT, many stress response genes were expressed in different patterns in the ON lines (Fig. 7a). According to the gene descriptions, we discovered that many of these differentially expressed genes are involved in drought and high salinity stress responses and may interact with the ABA signalling pathway. Some genes, including *LEA3*, some GTPases (*RAB21, RAB16C, RAB16D*), and sucrose nonfermenting1–related protein kinase2 (SnRK2) protein kinase genes (*SAPK1* and *SAPK10*), that function in ABA signalling and stress tolerance were significantly down-regulated in ON11 plants compared with the WT in the result of real-time PCR (Fig. 7b,c). These results verified the microarray data and revealed a potential relationship between *OsNAC2* and the ABA-dependent stress signalling pathway.

**Figure 8:**
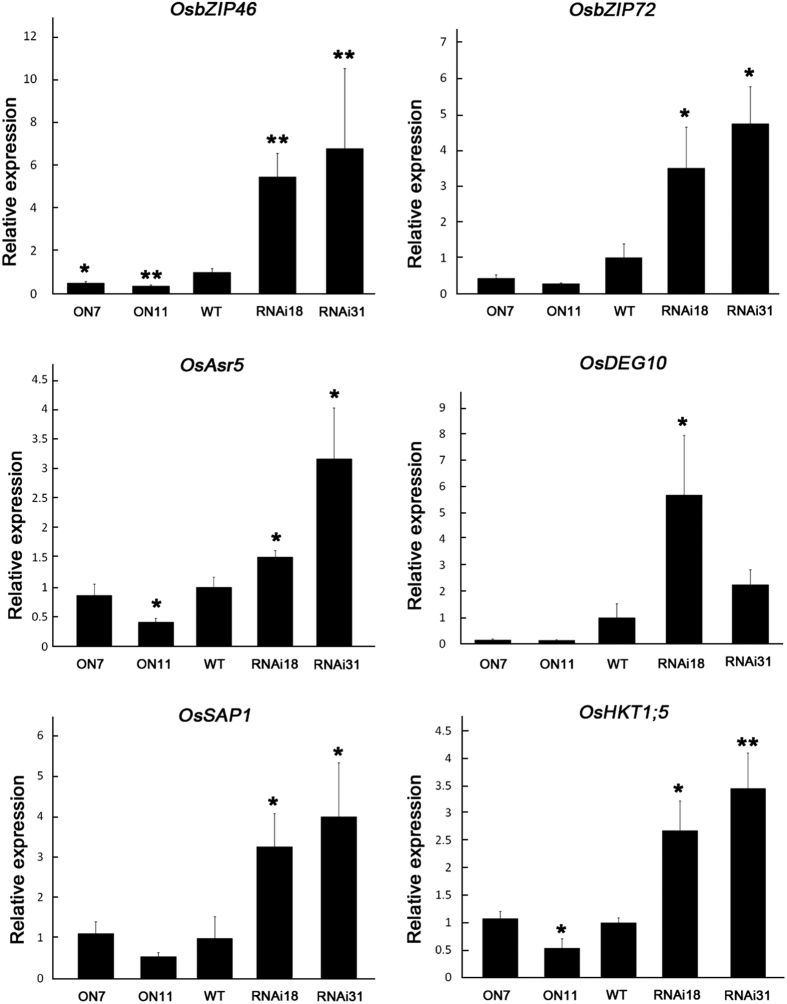
Expression analysis of stress-related and ABA signaling pathway genes in WT, OsNAC2-overexpressing plants and RNAi plants. The materials grew under normal conditions, 28 °C, 16 h light and 8 h dark. RNA was extracted from whole seedlings. Data were means ± SE with at least three biological replicates. Asterisks represent statistically significant differences between WT and transgenic plants. *P < 0.05, **P < 0.001, ***P < 0.001.

Based on previous reports and our data^40^,^41^, we examined two other ABA signalling pathway genes to confirm the relationship between *OsNAC2* and ABA signalling (Fig. 8). *OsbZIP46* and *OsbZIP72* are two members of the third subfamily of bZIP transcription factors in rice, which belong to the bZIP TF family and act downstream of ABA^42^. *OsbZIP46* and *OsbZIP72* are positive regulators of ABA signaling and drought stress tolerance of rice^40^,^41^,^43^. The higher transcription of these two genes in RNAi lines matches our previous result that RNAi lines had higher stress resistance. Besides, more stress-related genes are checked. *OsAsr5, OsDEG10, OsSAP1* and *HKT1*;5 are stress marker genes, which responds to ABA^44^,^45^, and participates in stress signaling^46^, stress tolerance^47^ and Na^+^ exclusion^48^. Those genes all showed up-regulated transcription in RNAi lines and might confer stress tolerance in RNAi lines. In conclusion, overexpression of OsNAC2 brings two major differences, one is altered ABA-signaling pathway, and the other is more severe stress phenotypes.

### Expression of the stress marker gene *OsLEA3* and SnRK2 protein kinase gene *OsSAPK1* are regulated by direct binding of OsNAC2 to their promoters

**Figure 9:**
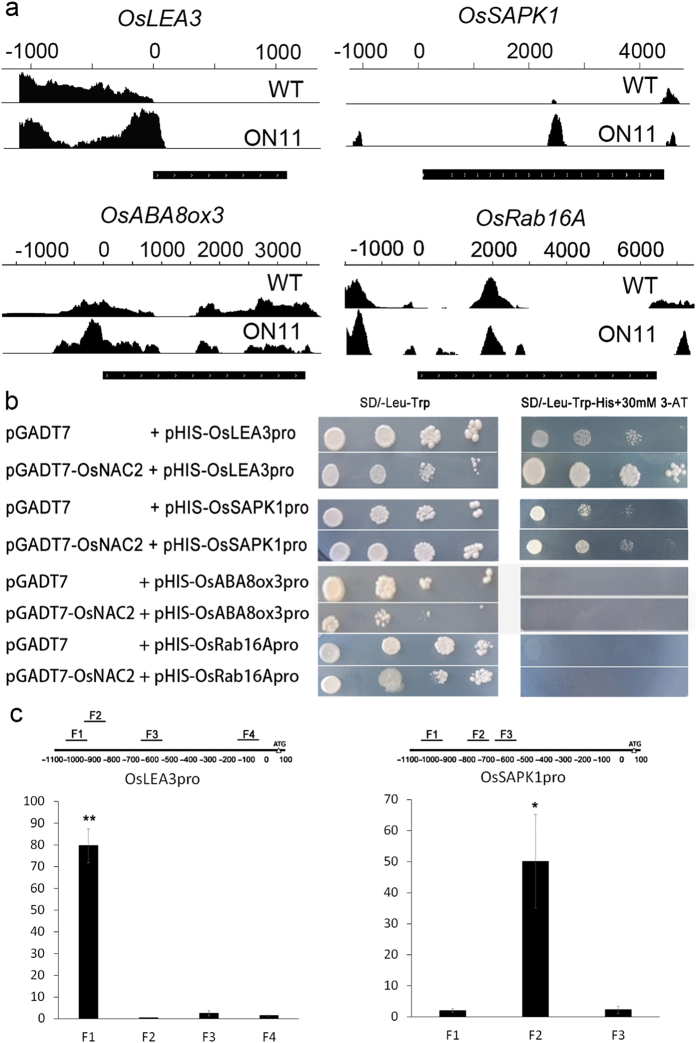
*OsLEA3* and *OsSAPK1* are the direct target genes of *OsNAC2*. (**a**) Binding peaks of *OsLEA3, OsSAPK1, OsABA8ox3* and *OsRab16A* in ChIP-seq assay. Black peaks represent for sequence hits on DNA of each gene regions. Higher the peak is, more binding in this region. The bars above the peak show the distance from ATG start codon of each gene. The black bar under the peak represents for the coding area of each gene, arrows on the bar show the coding direction of the gene. (**b**) Yeast one-hybrid assays showed that OsNAC2 Only binded to the promoter of *OsLEA3* and *OsSAPK1*. (**c**) ChIP-PCR assays. Total protein extracts from 35 S:OsNAC2–mGFP transgenic plants grown on MS-agar for 2 weeks were immunoprecipitated with an anti-GFP antibody. Fragmented genomic DNA was eluted from the protein–DNA complexes and subjected to qPCR analysis. The long black bars represent for promoter region which we designed primers for. The numbers under the bar show the distance from ATG start codon. Short bars represents for the corresponding region of each pair of primers on the promoter. Error bars are the standard error (SE) for three biological repeats, *P < 0.05, **P < 0.001.

We have shown that overexpression of OsNAC2 reduced plant tolerances to drought and high salinity stress. To identify the target genes of OsNAC2, we performed ChIP-seq where we scanned the promoter region of several stress-related and ABA-pathway genes. We found binding peaks in four of the candidate genes: *OsLEA3* (0 bp~−200bp and −900bp~−1100bp), *OsSAPK1* (−900bp~−1100bp)*, OsABA8ox3* (0 bp~−200bp), and *OsRAB16A* (−100~−250bp) (Fig. 9a). To further confirm whether these genes are direct targets of OsNAC2 we performed a yeast one-hybrid assay and ChIP-qPCR. Here we use *OsNAC2* overexpression line 11 (ON11) and WT (as negative control) for this assay. In transgenic line ON11, a GFP-tag was fused with *OsNAC2* CDS, which was promoted by 35 S. In the yeast one-hybrid assay, the coding region of the full-length *OsNAC2* cDNA was fused in frame to the GAL4 activation domain of the pGADT7 vector. Promoter sequence regions of *OsLEA3* (1063 bp), *OsSAPK1* (1080 bp)*, OsABA8OX3* (1090 bp) and *OsRab16A* (1090 bp) were ligated into the pHIS vector. The yeast one-hybrid assays suggested that OsNAC2 directly interacts with the promoter sequences of *OsLEA3* and *OsSAPK1* (Fig. 9b).

To test whether OsNAC2 specifically bound to its target genes, we performed ChIP-PCR experiments using anti-green fluorescent protein antibodies. We analysed the chromatin immunoprecipitated DNA for specific enrichment of the OsNAC2 target genes mentioned above. The specificity of the ChIP data was demonstrated using negative control DNA derived from WT plants (Fig. 9c). Together, these three data confirmed that OsNAC2 associates specifically with its target genes *OsLEA3* (−950bp~−1100bp) and *OsSAPK1* (−700~−850bp).

## Discussion

NAC family members function widely in plant growth and development processes, especially in relation to abiotic and biotic stress^49^,^50^,^51^. Most *NAC* genes have been reported to work as positive stress response TFs. For instance, *SNAC1* can greatly increase the drought and high salinity tolerance of rice by decreasing the transpiration rate^28^. OsNAC5 and OsNAC6 can directly bind to the promoter of *OsLEA3* and significantly promote high salt and drought tolerance^29^,^30^,^31^ while overexpression of *OsNAC10* increases rice yield under drought conditions in the flowering stage^32^.

In our research, *OsNAC2* expression was shown to be strongly induced by ABA and several abiotic stressors. The function of *OsNAC2* went exactly opposite to other reported *NAC* members like *OsNAC5, OsNAC6* and *OsNAC10*. We inferred that OsNAC2 might have new features and pathways in the regulation of abiotic stress in rice, which could enrich the biological functions and regulatory mechanisms of NAC family.

The heading stage, which can also be called flowering stage, is the most critical period in rice agricultural production. During this period rice plants are most sensitive to drought and grain yield is more severely affected by drought stress than at any other stage^52^. We performed drought stress experiments at the flowering stage in WT and transgenic plants to determine whether OsNAC2 influenced grain yield under drought conditions. The grain yield of WT and transgenic plants did not differ under normal cultural conditions. After drought stress at the flowering stage, however, RNAi plants showed a significantly higher seed setting rate. In terms of 1000-grain weight, RNAi lines also showed significantly higher grain yields than WT lines. In summary, various agricultural traits, such as the seed-setting rate and 1000-grain weight of RNAi lines were significantly higher than that in WT lines after drought treatment at the flowering stage. *SNAC1*^28^, *OsNAC5*^29^, and *OsNAC10*^32^ enhanced drought resistance in transgenic rice plants at the reproductive stage without affecting yield. Consistent with other NAC family members, therefore, we believe that *OsNAC2* represents a practical means of guiding rice agricultural production in drought or salt stress conditions.

Compared with WT plants, we found that many TF and stress-related genes were down-regulated in ON11 lines in our rice gene microarray chip. The expression levels of some of these genes were checked using qRT-PCR and this confirmed that all of these genes were down-regulated in ON11 lines. Many assays have reported that NAC family members can regulate stress-related gene expression resulting in transgenic rice plants with altered tolerance under different adverse conditions. ONAC045^49^ and OsNAC5^29^ can directly bind to the promoter region of *OsLEA3*. The *SNAC1* promoter contained two DREs, and ABA-responsive elements could be identified in the *OsNAC6* promoter^49^. Therefore, we hypothesise that *OsNAC2* may down-regulate these stress genes in response to abiotic stress.

Notably, most of the marker genes we tested were ABA-responsive genes. Some are ABA signalling pathway genes, such as *OsbZIP46*^40^ and *OsbZIP72*^41^. OsbZIP46, reported to be involved in stress resistance^40^, was phosphorylated by *OsSAPK2, OsSAPK6*, and *OsSAPK9*. Other ABA-responsive genes examined here are stress related genes that are up-regulated by ABA including *OsASR5*^45^, *OsDEG10*^46^, *OsSAP1*^47^ and *OsHKT1*;5^48^. We believe that OsNAC2 regulates abiotic stress through an ABA-dependent pathway by acting as a negative regulator of ABA and stress responsive genes. Additionally, we found that OsNAC2 can directly bind to the promoters of *OsLEA3* and *OsSAPK1* (Fig. 9) and down-regulate their expression (Fig. 7b,c). *OsLEA3* is a widely known stress marker gene, overexpression of which confers plant stress resistance^29^. *OsSAPK1* belongs to the SnRK2 family that can be upregulated by osmotic stressors such as salt and mannitol^53^. Here, we found that *OsSAPK1* expression can be directly regulated by OsNAC2 in response to abiotic stressors like drought and high salinity in rice.

To further investigate the biological function of *OsNAC2*, we constructed *OsNAC2*-overexpressing and RNAi lines. During cultivation we found several different phenotypes in the overexpression lines including shorter roots, shorter shoot lengths^38^ and premature leaf senescence (data not shown). This is consistent with evidence that plant tolerance to abiotic stress is often closely associated with growth and development. Root-specific overexpression of *OsNAC10* enlarges roots, enhancing the drought tolerance of transgenic plants and increasing their grain yield significantly under field drought conditions^32^. Conversely, *OsNAC5* can improve the stress tolerance of rice without affecting its growth^29^. Our results show that *OsNAC2* functions through the ABA-dependent pathway and may down-regulate many ABA-responsive stress marker genes. Previous work in our lab on *OsNAC2* has indicated that it functions in plant height development and potentially in root development (data not published), and in senescence (data not published). Combined with the results of this study, *OsNAC2* may be a powerful TF that serves as a node to link multiple pathways together.

In conclusion, we found that the expression of *OsNAC2* was induced by osmotic stress and ABA. Overexpression of *OsNAC2* resulted in transgenic rice plants that were sensitive to high salinity and drought stress at different growth stages. OsNAC2 directly down-regulated the stress-related marker gene *OsLEA3* and SnRK2 family gene *OsSAPK1* via the ABA-dependent pathway. Future work is required to determine the specific position of *OsNAC2* in the underlying regulatory network in more detail.

## Methods

### Plants, strain and plasmid

Wild type rice (*Oryza sativa*) which have an ecotype of Nipponbare, were saved in our lab. We constructed a vector in which 1500 bp full length promoter of *OsNAC2* is connected with *GUS* (Supplementary data Fig. 1). Overexpression and RNAi transgenic lines were constructed before and the details is in Chen *et al*.^38^. pCR-Blunt, pCAMBIA-1304 (containing a CaMV 35 S promoter), pCAMBIA1300 (fused with GUS reporter gene), RNAi vector, *Escherichia coli* strain DH5αand *Agrobacterium* strain EHA105 were all saved in our lab.

### Quantitative RT-PCR analysis

Total RNAs were extracted with TRIzol (TAKARA) from rice seedlings. All rice seedlings were grown under normal conditions of a 16 h light/8 h dark cycle at 28 °C for two weeks. The total RNAs were reverse-transcribed into first-strand cDNA using reverse-transcription enzyme. qRT-PCR was performed in a 10 *μ*L reaction with SYBR (Perfect Real Time code: DRR041 TaKaRa) 5 *μ*L, 10 mmol/L PCR forward primer 0.4 *μ*L, 10 mmol/L PCR reverse primer 0.4 *μ*L, template cDNA 4.2 *μ*L. The reaction used iCycleriQTM real-time quantitative PCR detection system (Bio-RAD). Each sample was repeated 3 times for qRT -PCR detection. We determined the linear range of the target gene and actin gene by detecting standard curve in each experiment. Osactin was used as an inner control. The relative expression levels of the gene were calculated by 2^−ΔΔCt^ analysis. Sequences of primer pairs are listed in supplemental data.

### Detection of histochemical GUS activity

Two-week-old rice seedlings were treated with 150 mM NaCl for 1, 2, 3, 4 and 5 days and with air dry condition for 0 h to 2 days. Tissues were then immersed in X-gluc solution in 37 °C overnight. Then the materials were soaked with 70% ethanol to remove chlorophyll. Wild type rice seedlings and tissues were used as normal control.

### Stress treatments of rice seedlings

For vegetative tolerance experiment, all rice seedlings were grown in basal nutrient solution and normal conditions, such as 28 °C, 16 h light and 8 h dark and then two-week-old seedlings were transferred into nutrient solution containing 150 mM NaCl for 2.5d or 20% PEG8000 for 5d and recovery for 2d and 3d respectively. For soil experiments, rice seedlings were grown under normal conditions for four weeks, and then irrigated with 150 mM NaCl solution for 14d or no water for 14d with 7d recovery.

### Physiological index measurement in high salt and drought tolerance experiments

The withered or survival rate, fresh weight, chlorophyll content and plant root or shoot length we mentioned were measured after stress or recovery. In withered or survival rate statistics, seedlings with all leaves yellow and wilted were calculated. DAB staining were performed after NaCl and PEG stresses. Rice leaves were immersed in DAB staining solution in 25 °C for 24 h, and then soaked with 95% ethanol to remove chlorophyll. Wild type rice seedlings and tissues were used as normal control. Electrolyte leakage was measured as described^54^. The F_v_/F_m_ values were determined using LI-6400XT portable photosynthesis system following with instructions. Rice leaves were immersed in the extract solution (70% acetone + 20% ethanol + 10% water) at 4 °C until the leaves were bleached. Chlorophyll content was detected using 721 visible light spectrophotometer. Agronomic traits were not counted until rice seeds turn mature.

### Yeast one-hybrid

For yeast one-hybrid assays, the coding sequence of *OsNAC2* was inserted into *Eco*R I*-Xho* I site of pGADT7 vector to generate a construct with activation domain and *OsNAC2*. The promoter sequence of *OsLEA3* (1063 bp), *OsSAPK1* (1080 bp)*, OsABA8OX3* (1090 bp) and *OsRab16A* (1090 bp) genes was inserted into pHIS2.1 vector through *Eco*R I*-Sma* I, *Eco*R I*-Mlu* I, *Eco*R I- *Mlu* I, *Eco*R I- *Mlu* I sites to generate an in-frame fusion with minHIS3. All primers used for cloning these constructs are listed in Table S2. These vectors and empty vector were transformed into yeast strain AH109 by the PEG/LiAc method. Yeast cells were plated onto SD/-His/-Trp/-Leu + 30 mM 3AT medium for stringent screening of the possible interactions, according to the protocol of Matchmaker GAL4 One-Hybrid System (Clontech, www.clontech.com).

### ChIP(Chromatin immunoprecipitation)-PCR and ChIP-seq

ChIP assay was performed based on the previous report^55^ with two-week ON11 transgenic seedlings, in which a mGFP coding sequence was fused in frame to the 3′ end of the *OsNAC2* gene in transgenic line, and the expression is driven by 35 S promoter. Because WT doesn’t have GFP, we use it as negative control to erase the background noise. Two-week-old seedlings grown in basal nutrient solution were treated for extraction of total proteins. The OsNAC2 protein was immunoprecipitated using an antibody against GFP. The DNA fragments of the ChIP were then used for quantitative PCR or sequencing. The ChIP-PCR experiments were repeated three times with the similar data. Primer pairs for qRT-PCR were listed in Table S2. For ChIP-seq, libraries were generated using Ovation Ultralow Library System 2 (Nugene) following manufacturer’s standard protocols. Sequencing was performed on a HiSeq2500 (Illumina) using pair end 100 bp mode.

## Additional Information

**How to cite this article**: Shen, J. *et al*. The NAC-type transcription factor *OsNAC2* regulates ABA-dependent genes and abiotic stress tolerance in rice. *Sci. Rep.* **7**, 40641; doi: 10.1038/srep40641 (2017).

**Publisher's note:** Springer Nature remains neutral with regard to jurisdictional claims in published maps and institutional affiliations.

## Supplementary Material

Supplementary Data
