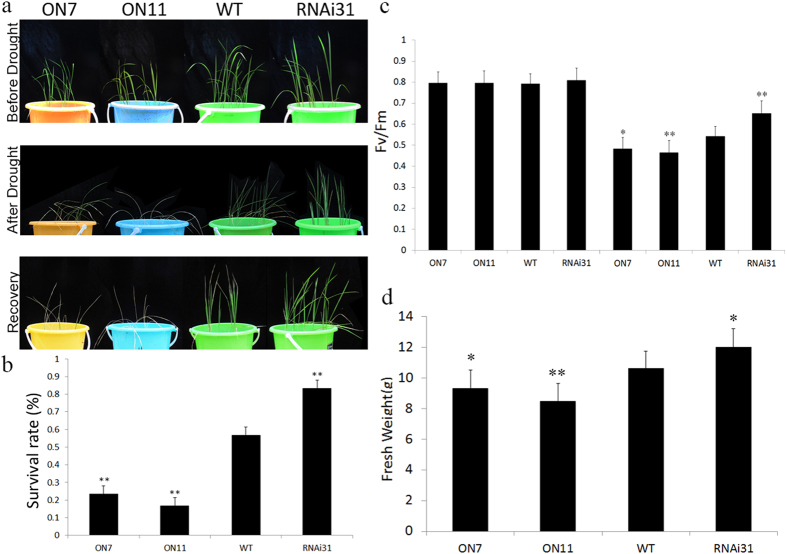# Corrigendum: The NAC-type transcription factor OsNAC2 regulates ABA-dependent genes and abiotic stress tolerance in rice

**DOI:** 10.1038/srep46890

**Published:** 2017-08-29

**Authors:** Jiabin Shen, Bo Lv, Liqiong Luo, Jianmei He, Chanjuan Mao, Dandan Xi, Feng Ming

Scientific Reports 7: Article number: 40641; 10.1038/srep40641 published online: 01 11 2017; updated: 08 29 2017.

This Article contains an error in Figure 5a, where the panel ‘After Drought’ is incorrect. The correct Figure 5 appears below as Figure 1.

## Figures and Tables

**Figure 1:**