# Supplementary material for: The NAC-type transcription factor OsNAC2 regulates ABA-dependent genes and abiotic stress tolerance in rice
Source: Sci Rep. 2017 Jan 11;7:40641. doi: 10.1038/srep40641 (PMC5225416; doi:10.1038/srep40641)
Supplement: Supplementary Data [file srep40641-s1.pdf]

The NAC-type transcription factor *OsNAC2* regulates ABA-dependent genes and abiotic stress tolerance in rice

Jiabin Shen[#1], Bo Lv[#1], Liqiong Luo[#1], Jianmei He[2], Chanjuan Mao[1], Dandan Xi[1], and Feng Ming[1,*]

1 State Key Laboratory of Genetic Engineering, Institute of Genetics, Institute of Plant Biology, School of Life Science, Fudan University, Shanghai 200433, China

2 Rice Research Institute, Sichuan Agricultural University, Sichuan 611130, China

[#] These authors contributed equally to this work.

[*]Corresponding authors: F Ming, Tel: 86-21-51630555; Email: fming@fudan.edu.cn

Supplemental data

Table S1: Primers used for realtime-PCR.

Gene names	Primer
OsNAC2	F: gagaagtctggctgggtcat<br>R: aacacccactcgttgttgga
OsACTIN	F: ctgcgataatggaactggt<br>R: acaatgctggggaagaca
OsLEA3	F: ggcgcagtacaccaagga<br>R: acctgctcactcgcctgt
OsRAB21	F: gagggaggagcacaagacc<br>R: attccatcatcctcagacgag
OsRAB16C	F:cagttccagccggtgaag<br>R: catcccgtcgtcctcaga
OsRAB16D	F: cgtctctccctgctttctga<br>R: ttcttcaacgggacgacaa
OsSAPK1	F: gccacagcgtatgatgcgta<br>R: taatctcttggccgctcgtg
OsSAPK10	F: tcttgacgtggaaagcagtg<br>R: tccacttcccagtttcttgg
OsbZIP46	F: atcaagaacagggagtccgc<br>R: gagccatcaccattcaccaa
OsbZIP72	F: aatgaggtagaagaaatgat<br>R: gcacagtcgctgatgaagg
OsASR5	F: aagcaagcaaagccacatct<br>R: cgtccttcttgtggtggaac
OsDEG10	F: agcggatcgacaagttattg<br>R: aacacaaccagcacctcatg
OsSAP1	F: acctgaggtccccttccat<br>R: tgccttaggtggtcagaacc
OsHKT1;5	F: acctgaggtccccttccat<br>R: tgccttaggtggtcagaacc

Table S2: Primers used for yeast one-hybrid and ChIP-qPCR.

Gene names	Primer
*OsNAC2*pro-F	5'- cggaattcgtacccctgattctaatcaggt -3'
*OsNAC2*pro-R	5'- cgggatccaagtgggaggggtggtggtgcc -3'
OsNAC2-F-*Eco*R Ⅰ	5'- cagaattcatggagcagcatcagggc -3'
OsNAC2-R-*Xho* Ⅰ	5'- aaactcgagttagtagccccatagcgcg -3'
OsLEA3-F-*Eco*R Ⅰ	5'- ggaattcctcatcctttgttgatctgtgg -3'
OsLEA3-R-*Sma* Ⅰ	5'- tcccccgggtgatcctaagcttcaaaaatta -3'
OsSAPK1-F-*Eco*R Ⅰ	5'- ggaattcacatccgatagttaaaactttg -3'
OsSAPK1-R-*Mlu* Ⅰ	5'- cgacgcgtatcccctcaacgacctcgctcc -3'
*OsABA8OX3-F-Eco*R Ⅰ	5'- ggaattctagcaaatcaacagggtatagt -3'
OsABA8OX3-R-Mlu Ⅰ	5'- cgacgcgtccaaatgaccctggagaattaa -3'
*OsRab16A-F-Eco*R Ⅰ	5'- ggaattctctcaaccttttaaactaagta -3'
OsRab16A-R-Mlu Ⅰ	5'- cgacgcgtgttggacctggatgtgcaaact -3'
*OsLEA3*pro-F1-F	5'- atccatgaggtagcatgcaagc -3'
*OsLEA3*pro-F1-R	5'- acgacgacgtatcgaacggccag -3'
*OsLEA3*pro-F2-F	5'- agctgccgtatctagcttcagg -3'
*OsLEA3*pro-F2-R	5'- gtggcgtggcatgctagaaaag -3'
*OsLEA3*pro-F3-F	5'- cgttcttgcttggctggtttac -3'
*OsLEA3*pro-F3-R	5'- aatgcacggacctctgttcttc -3'
*OsLEA3*pro-F4-F	5'- ccggagagagccgaagccgacg -3'
*OsLEA3*pro-F4-R	5'- gacaagagaagatgttcattta -3'
*OsSAPK1*pro-F1-F	5'- gtattaaacataggctaaaaaa -3'
*OsSAPK1*pro-F1-R	5'- tagtaacacactgtcaaatcat -3'
*OsSAPK1*pro-F2-F	5'- cggatctaaacaccccataagg -3'
*OsSAPK1*pro-F2-R	5'- gttaggtttggaggggattgag -3'
*OsSAPK1*pro-F3-F	5'- gcctaagggtctgtttagatcc -3'
*OsSAPK1*pro-F3-R	5'- acacgcaatctgtataattag -3'

Figure S1: Promoter sequence of OsNAC2 (Primer regions are underlined).

gtacccctgattctaatcaggttcagggcaaatgattatacgtactactgtacaatccctgcagaaaattacgcacaaag
accaaatatcaaggtttgctatggactgaatatatcttttgtttgcgcctggacgtcggtagctaccccagatattaattaa
ttatttgaattagtgattttgtaccaaatgatcgaaccagctcatgcttagcatgaagctaataaattctcccgtttattgca
gtactcgaatacaaatagtaggtgtactactcgccatggagtctgctctaattcgttctcaaaagcttctggagacgaagg
attggtttggtcgctttctcggttgctgctcggctcgcgtgtcgcatatgacgctttacttaatgctgctagtagtactcataa
tttccattaattaagactcatttaattagtaatgcaccaaatccttaaatccagccagatcagatcaaagtcatcctagggt
aaaacactctgatctgatggatcactagccactaaattaatccggtgattgatcatgtgtggaagcaagttttggctcgtt
acaaggtactattacataacactacgctgtcctgtatagctatctcatctatctcttcccatgcatttttcacctactagcta
gtactccatccgttttaaaatgtttgacaccgttgactttttagcacatgtttgaccgttcgtcttattcaaaaatttttgtga
aatatgtaaaactatatgtgtacatgaaagtatatttaacaataaatcgaatgatatgaaaagaataaataactacttaa
attttttgaataagacgaatggttaaacacgtactaaaaagtcaacggtgtcaaacattttgaaatggagggagtagtaa
cctgtgatgagtttccttcatcacccacgtctctttctactaaaaagaccatcatgtagctcgcctctagtatttaaggggca
ccaccacccctcccactttatttcctctatacaacgatttcctcttgtcaccctgaatctacttctgctgcaaaaggtaattagcatatcctaa
ctatttatcacctcatccctcaagtgtgttcttctactttcttagatgtactttttaaacttttctgtgcataaacatctcatgtgctgattctttttgtg
tgttctatgtgcttgcataccatgtccgtttggttcgattcgattttttaagcatccgtgtggtttgtttttgttctagcttagctagctagctagcaa
agaggtgtcatggatacataccagctttaatttgatttggtttgctctacctttaaaagtgtgtgaatcatctccggtcctagtttatgcatatctg
tgtgcatatgtacaaatgtttcaggacaggagttggcataacagctaacaaatcttacatatatagcagcaataagcaaggagcagttagcc
aggtaaagctctagctagctagcttaggcagca

Figure S2: OsNAC2 promoter structure and regulatory elements.

In *OsNAC2* promoter, there exist drought-response element and ABA-response element ABRE, salt-response element GT-1, Heat Shock element HSE, WRKY-binding element W-box, CGTCA-motif and TGACG-motif for MeJA responsiveness, ethylene-responsive element ERE, G-Box for light responsiveness, MBS for MYB binding site involved in drought-inducibility, gibberellin-responsive element P-box, TC-rich repeats for cis-acting element involved in defense and stress responsiveness, salicylic acid response element TCA-element, and circadian control element. This result is analyzed by PlantCARE.

OsNAC2 promoter analysis

```
+ GTACCCCTGA TTCTAATCAG GTTCAGGGCA AATGATTATA CGTACTACTG TACAATCCCT GCAGAAAATT
- CATGGGGACT AAGATTAGTC CAAGTCCCGT TTACTAATAT GCATGATGAC ATGTTAGGGA CGTCTTTTAA
                                                              TC-rich repeats
+ ACGCACAAAG ACCAAATATC AAGGTTTGCT ATGGACTGAA TATATCTTTT GTTTGCGCCT GGACGTCGGT
- TGCGTGTTTC TGGTTTATAG TTCCAAACGA TACCTGACTT ATATAGAAAA CAAACGCGGA CCTGCAGCCA

+ AGCTACCCCA GATATTAATT AATTATTTGA ATTAGTGATT TTGTACCAAA TGATCGAACC AGCTCATGCT
- TCGATGGGGT CTATAATTAA TTAATAAACT TAATCACTAA AACATGGTTT ACTAGCTTGG TCGAGTACGA

+ TAGCATGAAG CTAATAAATT CTCCCGTTTA TTGCAGTACT CGAATACAAA TAGTAGGTGT ACTACTCGCC
- ATCGTACTTC GATTATTTAA GAGGGCAAAT AACGTCATGA GCTTATGTTT ATCATCCACA TGATGAGCGG

+ ATGGAGTCTG CTCTAATTCG TTCTCAAAAG CTTCTGGAGA CGAAGGATTG GTTTGGTCGC TTTCTCGGTT
- TACCTCAGAC GAGATTAAGC AAGAGTTTTC GAAGACCTCT GCTTCCTAAC CAAACCAGCG AAAGAGCCAA
                     TGACG-motif                circadian
+ GCTGCTCGGC TCGCGTGTCG CATATGACGC TTTACTTAAT GCTGCTAGTA GTACTCATAA TTTCCATTAA
- CGACGAGCCG AGCGCACAGC GTATACTGCG AAATGAATTA CGACGATCAT CATGAGTATT AAAGGTAATT
                     CGTCA-motif                        circadian
+ TTAAGACTCA TTTAATTAGT AATGCACCAA ATCCTTAAAT CCAGCCAGAT CAGATCAAAG TCATCCTAGG
- AATTCTGAGT AAATTAATCA TTACGTGGTT TAGGAATTTA GGTCGGTCTA GTCTAGTTTC AGTAGGATCC
                    G-box
+ GTAAAACACT CTGATCTGAT GGATCACTAG CCACTAAATT AATCCGGTGA TTGATCATGT GTGGAAGCAA
- CATTTTGTGA GACTAGACTA CCTAGTGATC GGTGATTTAA TTAGGCCACT AACTAGTACA CACCTTCGTT

+ GTTTTGGCTC GTTACAAGGT ACTATTACAT AACACTACGC TGTCCTGTAT AGCTATCTCA TCTATCTCTT
- CAAAACCGAG CAATGTTCCA TGATAATGTA TTGTGATGCG ACAGGACATA TCGATAGAGT AGATAGAGAA

+ CCCATGCATT TTTCACCTAC TAGCTAGTAC TCCATCCGTT TTAAAATGTT TGACACCGTT GACTTTTTAG
- GGGTACGTAA AAAGTGGATG ATCGATCATGAGGTAGGCAAA ATTTTACAAA CTGTGGCAAC TGAAAAATC
      W box
+ CACATGTTTG ACCGTTCGTC TTATTCAAAA ATTTTTGTGA AATATGTAAA ACTATATGTGT ACATGAAAG
- GTGTACAAAC TGGCAAGCAG AATAAGTTTT TAAAAACACT TTATACATTT TGATATACACA TGTACTTTC
        MBS                TCA-element
```

```
+ TATATTTAAC AATAAATCGA ATGATATGAA AAGAATAAAT AACTACTTAA ATTTTTTGAAT AAGACGAAT
- ATATAAATTG TTATTTAGCT TACTATACTT TTCTTATTTA TTGATGAATT TAAAAAACTTA TTCTGCTTA
GT1-motif G-Box                                                   HSE
+ GGTTAAACAC GTACTAAAAA GTCAACGGTG TCAAACATTT TGAAATGGAG GGAGTAGTAAC CTGTGATGA
- CCAATTTGTG CATGATTTTT CAGTTGCCAC AGTTTGTAAA ACTTTACCTC CCTCATCATTG GACACTACT
         ABRE        G-box                      ERE
+ GTTTCCTTCA TCACCCACGT CTCTTTCTAC TAAAAAGACC ATCATGTAGC TCGCCTCTAGT ATTTAAGGG
- CAAAGGAAGT AGTGGGTGCA GAGAAAGATG ATTTTTCTGG TAGTACATCG AGCGGAGATCA TAAATTCCC

+ GCACCACCAC CCCTCCCACT TTATTTCCTC TATACAACGA TTTCCTCTTG TCACCCTGAAT CTACTTCTG
- CGTGGTGGTG GGGAGGGTGA AATAAAGGAG ATATGTTGCT AAAGGAGAAC AGTGGGACTTA GATGAAGAC

+ CTGCAAAAGG TAATTAGCAT ATCCTAACTA TTTATCACCT CATCCCTCAA GTGTGTTCTTC TACTTTCTT
- GACGTTTTCC ATTAATCGTA TAGGATTGAT AAATAGTGGA GTAGGGAGTT CACACAAGAAG ATGAAAGAA
     P-box
+ AGATGTACTT TTTAAACTTT TCTGTGCATA AACATCTCAT GTGCTGATTC TTTTTGTGTGT TCTATGTGC
- TCTACATGAA AAATTTGAAA AGACACGTAT TTGTAGAGTA CACGACTAAG AAAAACACACA AGATACACG

+ TTGCATACCA TGTCCGTTTG GTTCGATTCG ATTTTTTAAG CATCCGTGTG GTTTGTTTTTG TTCTAGCTT
- AACGTATGGT ACAGGCAAAC CAAGCTAAGC TAAAAAATTC GTAGGCACAC CAAACAAAAAC AAGATCGAA

+ AGCTAGCTAG CTAGCAAAGA GGTGTCATGG ATACATACCA GCTTTAATTT GATTTGGTTTG CTCTACCTT
- TCGATCGATC GATCGTTTCT CCACAGTACC TATGTATGGT CGAAATTAAA CTAAACCAAAC GAGATGGAA
       GT1-motif
+ TAAAAGTGTG TGAATCATCT CCGGTCCTAG TTTATGCATA TCTGTGTGCA TATGTACAAAT GTTTCAGGA
- ATTTTCACAC ACTTAGTAGA GGCCAGGATC AAATACGTAT AGACACACGT ATACATGTTTA CAAAGTCCT

+ CAGGAGTTGG CATAACAGCT AACAAATCTT ACATATATAG CAGCAATAAG CAAGGAGCAGT TAGCCAGGT
- GTCCTCAACC GTATTGTCGA TTGTTTAGAA TGTATATATC GTCGTTATTC GTTCCTCGTCA ATCGGTCCA
                                                                      MBS
+ AAAGCTCTAG CTAGCTAGCT TAGGCAGCA
- TTTCGAGATC GATCGATCGA ATCCGTCGT
```

Motifs function：

ABRE：cis-acting element involved in the abscisic acid responsiveness

GT1-motif：light responsive element

HSE：cis-acting element involved in heat stress responsiveness

W box

CGTCA-motif：cis-acting regulatory element involved in the MeJA-responsiveness

ERE：ethylene-responsive element

G-Box：cis-acting regulatory element involved in light responsiveness

MBS：MYB binding site involved in drought-inducibility

P-box：Gibberellin-responsive element

TC-rich repeats：cis-acting element involved in defense and stress responsiveness

TCA-element：cis-acting element involved in salicylic acid responsiveness
TGACG-motif：cis-acting regulatory element involved in the MeJA-responsiveness
Circadian：cis-acting regulatory element involved in circadian control
